# 18-Year Monitoring of the Steno-Endemic *Verbascum rupicola* (Scrophulariaceae): Compounding Pressures and the Extinction Vortex

**DOI:** 10.3390/plants15101555

**Published:** 2026-05-20

**Authors:** Volkan Eroğlu

**Affiliations:** Department of Biology, Science Faculty, Ege University, Bornova, İzmir 35100, Turkey; volkan.eroglu@ege.edu.tr

**Keywords:** steno-endemic, habitat fragmentation, stochastic events, reproductive squeeze, germination kinetics

## Abstract

The steno-endemic *Verbascum rupicola* faces a precarious future due to its extreme habitat specialization on tectonically active hydrothermal quartz veins. This study presents a long-term assessment based on periodic population censuses spanning 18 years (2007, 2016, and 2025) to assess the demographic and spatial trends of its global population in the Tahtalı Dam basin, Türkiye. Field surveys, GIS-based habitat mapping, and controlled pollination experiments were integrated with seed germination kinetics and ex situ cultivation trials. Results reveal a precipitous 69.12% global population decline, primarily driven by a 33.41% habitat loss from agricultural expansion in 2011 and the total extirpation of three sub-populations by a major wildfire in 2017. Furthermore, a “reproductive squeeze” was identified, where climate-induced reductions in flower production (18.87%) are compounded by intensifying floral predation by *Pieris rapae*. Reproductive analysis revealed random monomorphic enantiostyly—reported for the first time in the genus—which functions as a flexible mating system prioritizing outcrossing while providing reproductive assurance. Despite high intrinsic seed viability (69.12%), ex situ cultivation largely failed (3.5% survival; 1 out of 28 transplanted individuals), underscoring the species’ obligate chasmophytic nature. Consequently, *V. rupicola* meets the criteria for Critically Endangered (CR) status, necessitating urgent “micro-reserve” protection of its remaining habitat and in situ restoration efforts.

## 1. Introduction

The Mediterranean Basin, encompassing diverse terrains from coastal dunes to montane refugia across southern Europe, North Africa, and Western Asia, represents a globally significant reservoir of plant biodiversity [1]. Spanning just 1.6% of Earth’s land surface, this region supports over 25,000 vascular plant species—approximately 10% of the world’s total [2]. This exceptional richness stems from a confluence of biogeographic crossroads and paleoclimatic oscillations, such as Pleistocene glaciations, which have both shaped and pressured the flora [3]. Endemic species, often narrowly distributed in isolated hotspots such as islands and mountain ranges, underscore the Basin’s role as a tension zone for evolutionary divergence, where ancient relict lineages coexist with recently speciated neo-endemics [4].

Despite its ecological importance, this biodiversity faces an intensifying crisis marked by habitat degradation, anthropogenic transformation, and climate-driven shifts [5,6,7]. Steno-endemic species, characterized by extremely restricted distributions and specific habitat requirements, are particularly vulnerable to these pressures [8]. The interplay between habitat fragmentation and stochastic environmental events, such as wildfires, can push these small populations into an “extinction vortex,” where genetic, demographic, and environmental factors reinforce each other to accelerate a species’ decline [9]. According to the IUCN, 76,864 plant species are in threatened status, and 6445 of these are in the critically endangered (CR) category [10]. Such alarming figures highlight an urgent need for longitudinal studies that integrate spatial and reproductive data to develop effective conservation strategies [11].

Among the myriad threats to steno-endemics, habitat fragmentation, climate change, and fires emerge as dominant drivers, each capable of disrupting plant population dynamics through isolation, physiological stress, and direct mortality [12,13]. An extinction vortex describes a self-reinforcing cycle of decline where initial perturbations trigger genetic, demographic, and ecological feedbacks that accelerate species loss, often culminating in local or global extinction [14,15]. For plants, these processes are particularly insidious in fragmented landscapes, where dispersal limitations compound the challenges of tracking shifting climates or surviving recurrent fires [16].

While individual stressors have been extensively studied, their combined impacts on plants reveal emergent synergies that individual analyses fail to capture [17]. For instance, fragmentation not only reduces habitat patches but also alters microclimates and fire propagation, while climate change intensifies fire frequency and severity, potentially overwhelming species’ regenerative capacities [18]. Yet, systematic syntheses of how these disasters interact to initiate and sustain extinction vortices remain scarce, particularly for plants across diverse biomes [19,20]. This gap hinders proactive conservation, as current strategies often address threats in isolation, underestimating the multiplicative risks in fire-prone or warming regions [21].

The genus *Verbascum* Linnaeus [22] (Scrophulariaceae) is a diverse lineage comprising approximately 491 species worldwide [23,24,25]. Türkiye serves as the primary center of diversity for the genus, harboring 255 species, of which 200 are endemic to the country [26,27,28,29]. This high endemism rate (78.4%) underscores the evolutionary significance of the Anatolian diagonal and the Mediterranean enclave for the diversification of the genus. Within this rich taxonomic framework, *Verbascum rupicola* (Hayek & Siehe) Hub.-Mor. [30] represents an extreme case of steno-endemism. Restricted to a single global locality in the Tahtalı Dam basin of İzmir, the species is an obligate chasmophyte known only from very limited rocky outcrops [31]. Despite its unique ecological positioning, *V. rupicola* has remained entirely unstudied since its taxonomic description, with no prior investigations into its population dynamics, reproductive biology, or habitat requirements. In the absence of any empirical data for *V. rupicola*, the potential existence of specialized floral morphologies and their role in mitigating or exacerbating the ‘reproductive squeeze’ under changing climatic conditions remains a critical unknown. Consequently, there is an urgent need for an integrated, long-term study to characterize the factors preventing the expansion of this species and to establish a baseline for its conservation.

In light of these research gaps, the present study provides the first comprehensive investigation of *V. rupicola* through periodic population censuses spanning an 18-year period (2007, 2016, and 2025). The primary objectives are to: (i) assess the demographic and spatial trends of the global population in response to habitat fragmentation and stochastic fire events; (ii) investigate the reproductive biology of the species, specifically exploring the presence of specialized floral morphologies such as enantiostyly; (iii) determine the impact of climatic variables and biotic pressures on reproductive success; (iv) evaluate the effectiveness of some ex situ conservation strategies; and (v) perform a formal conservation assessment of the species according to the IUCN Red List Categories and Criteria based on the generated demographic and spatial data. By integrating multi-layered ecological and reproductive data, this research seeks to clarify the factors driving the potential extinction vortex of this steno-endemic and to propose science-based conservation interventions for its long-term survival.

## 2. Results

### 2.1. Distribution Area and Population

#### 2.1.1. Habitat Specialization

**Figure 1:**
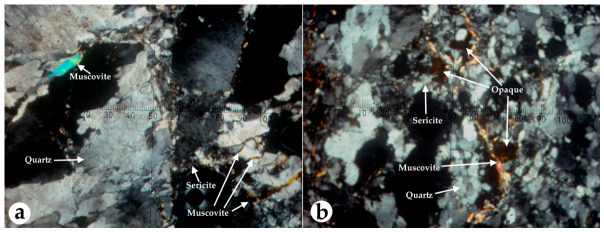
Petrographic analysis of the *V. rupicola* habitat substrate. (**a**) Microphotographs showing the hydrothermal quartz vein matrix with undulose extinction and tectonic orientation of muscovite and sericite, (**b**) alongside opaque mineral phases (pyrite/goethite) filling fractures (right).

Field observations and microscopic analyses indicate that *V. rupicola* is exclusively associated with tectonically active quartz systems. The host rock is identified as a hydrothermal quartz vein within quartzite, characterized by brown iron-oxide staining and localized mica flakes. Detailed petrographic examination revealed that the substrate consists of quartz crystals categorized into fine (5–110 µm), medium (100–1850 µm), and coarse (2 mm to cm) grains. A significant majority of these crystals exhibit undulose extinction, a definitive indicator of exposure to high tectonic pressure. The mineralogical composition includes muscovite and sericite aligned with tectonic orientation, along with trace amounts of rutile and anatase. The physical environment is defined by extreme hardness, reaching 7 on the Mohs scale in quartz-rich sections. Ore petrography confirmed an opaque phase dominated by fine-grained pyrite (45 × 50 µm) and secondary limonite (goethite) filling the fractures (Figure 1). The restriction of *V. rupicola* to these specific, tectonically deformed quartz veins showed a strict geological specialization unique to this species’ habitat.

#### 2.1.2. Spatial Habitat Contraction

**Figure 2:**
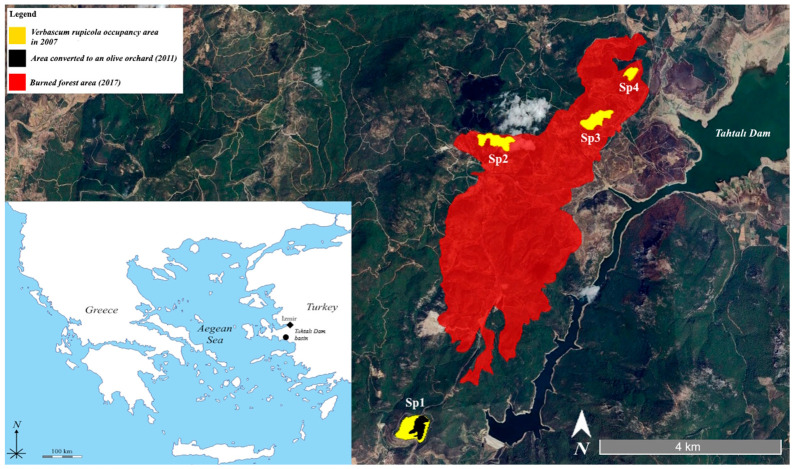
Spatiotemporal distribution and habitat loss of the steno-endemic *V. rupicola* in the Tahtalı Dam basin (2007–2025).

The initial baseline mapping in 2007 revealed a total global Area of Occupancy (AOO) of 623,765 m^2^ for *V. rupicola*, distributed across four distinct sub-populations. Sp1 was identified as the primary habitat (298,801 m^2^), followed by Sp3 (142,433 m^2^), SP2 (134,621 m^2^), and Sp4 (47,919 m^2^). Spatial analysis of land-use changes indicated significant habitat fragmentation and loss over the monitoring period. In 2011, agricultural expansion within Sp1 resulted in the conversion of 99,828 m^2^ of natural rocky habitat into olive orchards, representing a 33.41% reduction in Sp1’s original extent (Figure S1). Furthermore, the 2017 wildfire, which impacted an estimated 14,001,526 m^2^ in the Tahtalı Dam basin, completely engulfed the spatial boundaries of Sp2, Sp3, and Sp4. Consequently, by 2025, the global range of the species contracted to a single remnant patch within Sp1, totaling 198,973 m^2^. This constitutes a cumulative global habitat loss of 68.10% in 18 years (Figure 2).

#### 2.1.3. Demographic Trends and Population Collapse

The census data showed a catastrophic decline in the number of individuals across the entire range (Table 1). In 2007, the total global population consisted of 1250 mature individuals (Sp1: 810; Sp2: 183; Sp3: 193; Sp4: 64). The 2025 census revealed the total extinction of sub-populations SP2, SP3, and SP4 following the 2017 wildfire event. In the surviving Sp1 fragment, the population size dropped from 810 to 386 individuals, a 52.35% decrease within this specific sub-population. Globally, the species experienced a 69.12% demographic collapse between 2007 and 2025.

### 2.2. Impact of Climate on Reproductive Effort

A progressive decline in the reproductive output of *V. rupicola* was recorded over the 18-year monitoring period, as confirmed by an ordinary one-way ANOVA (*F*(2, 297) = 4.534, *p* = 0.0115). The mean flower number per individual fell significantly from 128.53 ± 73.92 in 2007 to 104.27 ±65.29 in 2016, reaching its lowest recorded value of 102.07 ± 67.37 in 2025. To further elucidate the drivers of this decline, a Multiple Linear Regression (MLR) model was constructed using 300 individual observations. The model (Flower Number = −235.9 + 18.54 × Temp + 1.807 × Rain) indicated that floral production is significantly influenced by both temperature (*p* = 0.0371) and precipitation (*p* = 0.0043) patterns. The predictive power of the model was verified with a Variance Inflation Factor (VIF) of 4.293, confirming that the results were not compromised by multicollinearity between climatic variables. While both factors are influential, the stronger statistical significance of rainfall suggests that the sharpest declines in flower production are tightly coupled with the increasing water stress observed in the Tahtalı Dam basin. These results confirm that the transition toward a more arid climate is directly suppressing the species’ reproductive capacity.

### 2.3. Impact of Climate on Predation

Parallel to the decrease in flower production, floral predation by *Pieris rapae* larvae showed a steady increase (Figure S2). The percentage of consumed flowers rose from 8.02% ± 23.22 in 2007 to 11.60% ± 26.6 in 2016, peaking at 12.48% ± 27.81 in 2025. The MLR results for predation (Predation Rate = 60.91 − 2.619 × Temp − 0.2788 × Rain) highlighted a negative correlation with rainfall (β = −0.2788). This indicates that as water availability decreased—particularly evident in the transition from 2016 to 2025—the predation pressure on the remaining floral resources intensified. The results suggest that the combination of reduced reproductive investment and increased herbivory is creating a critical bottleneck for the species’ persistence.

Contrary to the declining trend observed in floral production, the predation pressure on *V. rupicola* remained statistically stable over the 18-year monitoring period, as indicated by a one-way ANOVA (*F*(2, 297) = 0.8301, *p* = 0.4370). While the mean predation rate rose numerically from 8.02% ± 23.22 in 2007 to 12.48% ± 27.81 in 2025, this fluctuation did not reach statistical significance. The MLR analysis further confirmed that predation intensity is independent of the shifting climatic regime (*p* = 0.4370). Neither mean temperature (*β* = −2.626, *p* = 0.4313) nor total rainfall (*β* = −0.2793, *p* = 0.2381) significantly predicted the variation in predation rates. These results indicate that while herbivory exerts a constant biological pressure on the population, it has not escalated in response to the increasing aridity or thermal stress observed in the study area. Therefore, the critical bottleneck for the species’ persistence is not a climate-driven surge in herbivory, but rather the direct physiological suppression of reproductive output caused by environmental stressors.

### 2.4. Style Morphs and Enantiostyly

A total of 2742 flowers from 50 individuals were observed to assess style morph frequencies at the population level. The distribution consisted of 915 left-tilted (33.37%), 902 right-tilted (32.90%), and 925 straight-styled (33.73%) flowers. All three floral morphs were present within the same individuals and inflorescence axes randomly, indicating that *V. rupicola* exhibits random monomorphic enantiostyly. The Chi-square goodness-of-fit test confirmed that the observed frequencies did not significantly deviate from the expected 1:1:1 ratio (χ^2^ = 0.5709, df = 2, *p* = 0.7517).

### 2.5. Mating System

**Figure 3:**
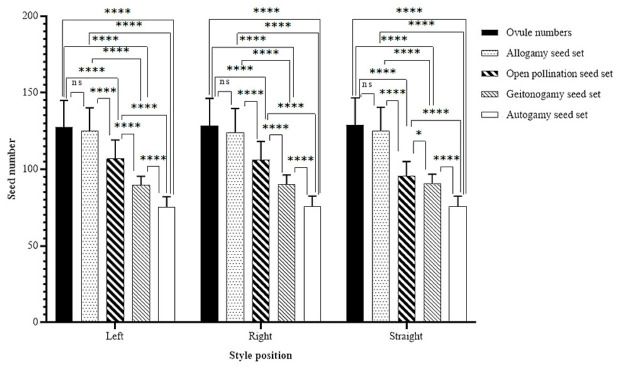
Comparison of reproductive system success among left-, right- and straight-styled flowers. Data were analyzed using Linear Mixed-Effects Model (LMM) with individual plant as a random factor, followed by Tukey’s multiple comparisons test. Asterisks indicate levels of statistical significance [ns (not significant),* (*p* < 0.05) **** (*p* < 0.0001)].

Experimental pollination treatments revealed that *V. rupicola* possesses a flexible reproductive system characterized by varying levels of seed set across different pollination modes (Figure 3). The mean number of ovules per capsule remained consistent across all floral morphs (left, right, and straight styled), ranging from 127.4 to 129.0. No significant differences were observed between the total ovule number and the seed set resulting from allogamy in any style position (*p* > 0.9). Across all style orientations, the highest reproductive success was achieved through manual cross-pollination (allogamy), yielding between 124.1 and 125.1 seeds per capsule. While open pollination followed as the next most successful treatment, it resulted in a significantly lower seed set compared to allogamy across all groups (*p* < 0.0001). This reduction, which was most pronounced in flowers with straight styles (Mean Diff = 24.16, *p* < 0.0001). In contrast, the apomixis treatment yielded no seeds in any of the trials, confirming that the species is not apomictic.

The species demonstrated a significant capacity for self-compatibility, although at reduced levels compared to outcrossing. Both geitonogamy and autogamy treatments produced seeds, but these yields were significantly lower than those from allogamy and open pollination (*p* < 0.0001). Geitonogamy yielded approximately 89.60–90.56 seeds, whereas the lowest seed production was recorded in the autogamy group (75.08–75.98 seeds). The further significant reduction in seed set from geitonogamy to autogamy (*p* < 0.0001) indicates that geitonogamy enhances reproductive success compared to autonomous selfing.

Linear Mixed-Effects Model (LMM) results confirmed that style orientation (left, right, or straight) did not fundamentally alter the hierarchy of the mating system, as the decline in seed set from allogamy to autogamy followed a highly consistent and significant pattern in all three morphs. However, the distinct advantage of open pollination over geitonogamy varied according to floral morphs. While this difference was highly significant in left and right-styled flowers (*p* < 0.0001), the margin of success was notably reduced in straight-styled flowers (*p* = 0.0139).

The Index of Self-Incompatibility (ISI) was determined as 0.601 for left styled, 0.609 for right styled, and 0.607 for straight styled flowers. These values consistently fall within the range of >0.2 and <1, identifying *V. rupicola* as a partially self-incompatible species across all floral morphs.

### 2.6. Seed Viability, Germination and Seedling Establishment

The germination of *V. rupicola* seeds at a constant 20 °C began on the second day, with the most rapid increase in cumulative germination occurring between days 3 and 5. The process reached a definitive plateau by day 8, yielding a mean germination percentage (GP) of 68.4% ± 12.6 (range: 54–88%). To mathematically characterize this performance, a non-linear regression model showed a high goodness-of-fit (*R*^2^ = 0.8845), estimating a maximum germination capacity (Top) of 69.12% (95% CI: 65.67–72.88%), a HillSlope of 0.4206, and a Log*EC*_50_ value of 3.770. These kinetic results were validated by TTC assays, which indicated a mean seed viability of 65.4% ± 8.7. Statistical analysis using a paired *t*-test confirmed no significant difference between the number of viable and germinated seeds (*t* = 1.337, df = 9, *p* = 0.2140), and a very strong positive correlation (*r* = 0.9482, *p* < 0.0001) was observed between viability and germination performance. These results confirm that the seeds exhibit high physiological quality, which enables virtually all viable seeds to complete germination without significant physiological dormancy.

The transition of *V. rupicola* from in vitro to substrate-based conditions revealed significant physiological sensitivity. In the controlled growth chamber, seedling survival decreased from 60 to 36 individuals in the first week, ultimately resulting in 28 viable seedlings (46.6% survival rate) by the end of the three-week acclimatization period. At this stage, the surviving seedlings exhibited healthy development, having reached the targeted 4–8 true leaf stage. The subsequent transfer to the outdoor rock garden led to a further decline in survival rates, reflecting the species’ specialized ecological requirements. Survival dropped from 28 transplanted seedlings to 12 within the first week, and only one individual (3.5% of the n = 28 transplanted group) remained viable beyond the third week. This surviving individual showed successful development and proceeded to flower 2.5 months after transplantation (Figure S3). However, the plant died shortly after the flowering phase. This outcome suggests that while the species can complete its phenological cycle under semi-natural conditions, it exhibits a critical inability to maintain long-term survival and perenniality outside of its specific native habitat.

### 2.7. Conservation Status and IUCN Red List Assessment

Based on the observations conducted over 18 years (2007–2025), *V. rupicola* is categorized as Critically Endangered (CR). The species exhibits an extremely restricted distribution, with an Extent of Occurrence (EOO) of less than 1 km^2^ and an Area of Occupancy (AOO) of 0.199 km^2^. Since the 2017 wildfire led to the total extirpation of three sub-populations, the species is now confined to a single remaining location (Sp1), where all 386 surviving mature individuals are concentrated. The documented 69.12% collapse in the total population size and the 33.41% reduction in habitat quality within the last remaining patch provide clear evidence of a continuing decline. These quantitative metrics directly satisfy the requirements for CR status under the criteria for restricted range and small, declining population structure. The detailed justification and the transparent calculation path for the assigned IUCN categories—B1ab(i, ii, iii, iv, v) + 2ab(i, ii, iii, iv, v)—are summarized in Table 2.

## 3. Discussion

The 18-year longitudinal monitoring and reproductive analysis of *Verbascum rupicola* (2007–2025) provides a compelling illustration of the synergistic threats confronting steno-endemic species in the Mediterranean Basin. Our findings reveal a rapid demographic and spatial decline consistent with an extinction-vortex trajectory, driven by a combination of anthropogenic transformation, stochastic environmental disturbances, and climate-induced reproductive stress.

### 3.1. Habitat Fragmentation and Legislative Gaps

The primary spatial bottleneck for the species originated in 2011 with agricultural expansion in Sp1. The conversion of 33.41% of the maquis shrubland in Sp1 into olive groves resulted in the fragmentation of the rocky substrates inhabited by *V. rupicola*, leading to a disproportionate 52.35% decline in this subpopulation. This land-use change is rooted in Article 2/B of the Forest Law No. 6831 of the Republic of Türkiye, which classifies maquis areas as land that has “lost its forest character,” thereby opening them to agricultural exploitation. Such legislative frameworks have led to the extensive destruction of not only maquis but also existing forest lands for agricultural growth [32,33]. Paradoxically, Türkiye has been a party to the Convention on Biological Diversity (CBD) since 1992, which explicitly mandates the protection of biodiversity, including plant species [34]. The destruction of a significant portion of the rocks in Sp1—identified by geologists as the “Bornova Complex” [35] and characterized by our findings as hydrothermal quartz veins formed by tectonic activity— would bring the species closer to the extinction vortex.

### 3.2. Fire Vulnerability and Metapopulation Collapse

Steno-endemic species with restricted distributions and weak post-fire recovery strategies face heightened extinction risks from high-intensity or recurrent wildfires that disrupt small populations in marginal habitats [36,37,38]. These species often lack serotiny or robust resprouting capabilities, rendering them more vulnerable than their widespread fire-adapted relatives [39]. Field surveys in 2025 revealed that populations in Sp2, Sp3, and Sp4, which were within the perimeter of the 2017 wildfire, had disappeared. This underscores *V. rupicola* as a fire-vulnerable species. The loss of 54.32% of the total population due to fire has not only depleted numerical abundance but also caused the collapse of metapopulation dynamics, making global survival dependent on a single fragmented site. In the loss of metapopulations, even if the species persists demographically in surviving patches, irreplaceable alleles and adaptive potential vanish along with the extirpated individuals [40,41,42]. In fragmented systems, maintaining gene flow through genetic rescue translocations to affected sites is crucial to counteract genetic drift and genetic load [43,44,45,46]. Consequently, we argue that seed translocation from Sp1 to the rocky substrates previously occupied by fire-extirpated individuals, coupled with monitoring of translocation success, is critical for future conservation efforts.

### 3.3. Climate-Induced Reproductive Squeeze

Beyond spatial and genetic constraints, our findings reveal an alarming trend of “Climate-Induced Reproductive Squeeze.” The strong positive correlation between precipitation and flower production indicates that *V. rupicola* is highly sensitive to the increasing aridity of the Aegean region. Record-low rainfall in 2025 limited the plant’s ability to invest in reproductive structures, resulting in an 18.87% decrease in flower abundance compared to 2007 levels. This vividly demonstrates the impact of global warming in driving the species toward an extinction vortex. Rising temperatures and decreasing precipitation have been reported to advance flowering and fruiting phenology, leading to reduced reproductive output [47]. Furthermore, in perennial species, drought conditions—conditioned by photoperiod and precipitation—have been shown to reduce flower and fruit yield [48].

This abiotic stress is exacerbated by intensifying asymmetric biotic pressure. The increase in predation rates by *Pieris rapae* caterpillars on *V. rupicola* flowers and fruits (rising from 8.02% in 2007 to 12.48% in 2025) further intensifies the reproductive burden on individuals already producing fewer flowers due to warming. In this “reproductive squeeze,” the species is caught in a pincer: while drought reduces flower production, the primary predator consumes a disproportionately higher percentage of the remaining reproductive units. This 55% increase in predation significantly reduces net seed rain, obstructing the natural recruitment of the seed bank onto rocky substrates and undermining the primary mechanism for demographic recovery. This highlights the integration of biological factors into the species’ extinction vortex. Studies have emphasized that climate-driven increases in predation rates are effective across many plant taxa, generally increasing the damage caused by herbivores depending on the insect group and drought severity [49,50,51,52].

### 3.4. Reproductive Strategy: Enantiostyly and Mating Success

In response to these multi-layered threats, our analyses of reproductive success, germination kinetics, and ex situ cultivation reveal a profound gap between the biological capacity of *V. rupicola* and its ecological reality. Our reproductive system analysis revealed the phenomenon of enantiostyly (right-, left-, and straight-tilted style morphs), reported here for the first time in the genus *Verbascum*. The balanced distribution of styles observed in the population indicates that the species exhibits “random monomorphic enantiostyly.” This type of floral asymmetry is emphasized as an advanced mechanism to minimize anther-stigma interference (sexual interference), particularly in species with nectar guides that direct pollinators to specific positions [53,54].

The prominent nectar guides in *V. rupicola* flowers may serve as critical cues that could standardize the pollinator’s entry direction and body position. Structures that channel pollinator behavior in this manner are expected to optimize the mechanical function of enantiostyly, potentially facilitating precise pollen deposition or removal from specific regions of the pollinator’s body [54,55]. Our experimental results showed that asymmetric (left and right) morphs achieved significantly higher success in open pollination compared to geitonogamy, suggesting the functional efficiency of stylistic deflection in capturing outcrossed pollen. As stated by Jesson and Barrett [56], the deflection of the stigma from the central axis prevents physical blockage by the flower’s own pollen (pollen clogging) while maintaining pollinator contact.

The most striking finding regarding the reproductive system of *V. rupicola* is the low level of statistical difference between open pollination and geitonogamy success in straight-styled flowers (*p* = 0.0139). Although nectar guides potentially direct the pollinator along a central path, the location of the stigma on this same axis in straight morphs suggests that sexual interference is more probable. This may lead to stigma saturation with self-pollen (allogamous) rather than outcrossed pollen [55,56]. While this does not necessarily lower the outcrossing potential of straight morphs, it biologically suggests that asymmetric morphs may act as a more selective “filter” for cross-pollination. Ultimately, the balanced morph ratio in *V. rupicola* is a stable strategy supporting disassortative mating to maintain genetic diversity. This flexible system prioritizes outcrossing while providing reproductive assurance through selfing when necessary.

### 3.5. Ex Situ Limitations and Conservation Implications

Analyses of seed viability and germination kinetics confirm that *V. rupicola* possesses high intrinsic reproductive potential. The 69.12% cumulative germination success achieved in the laboratory, which statistically aligns with TTC viability tests (*p* = 0.2140), proves that the extinction process cannot be explained by seed sterility or physiological dormancy. Instead, the rapid germination response (T_50_ = 5.89 days) suggests an “opportunistic” strategy evolved to exploit the limited precipitation windows of the Mediterranean climate.

Despite this laboratory success, the failure of ex situ cultivation efforts indicates an extremely narrow ecological niche. Low survival rates in seedlings transferred to soil and an extreme failure rate in the outdoor rock garden (only 3.5%, 1 individual) suggest that *V. rupicola* exhibits extreme ecological specialization as an obligate chasmophyte. Notably, the death of the sole survivor after flowering—contrary to the plant’s perennial nature—suggests that its phenological cycle is entirely dependent on the specific mineralogical and tectonic structures of its habitat, sensitive to microclimate, or that standard ex situ protocols fail to replicate necessary cues. Failures in the ex situ conservation of rare plants due to habitat specificity are well-documented; despite high germination in controlled environments, cultivation or reintroduction often fails because artificial conditions cannot mimic natural microhabitats, including soil type, hydrology, edaphic factors, or climatic cues [57,58,59].

## 4. Materials and Methods

### 4.1. Plant Species and Study Area

**Figure 4:**
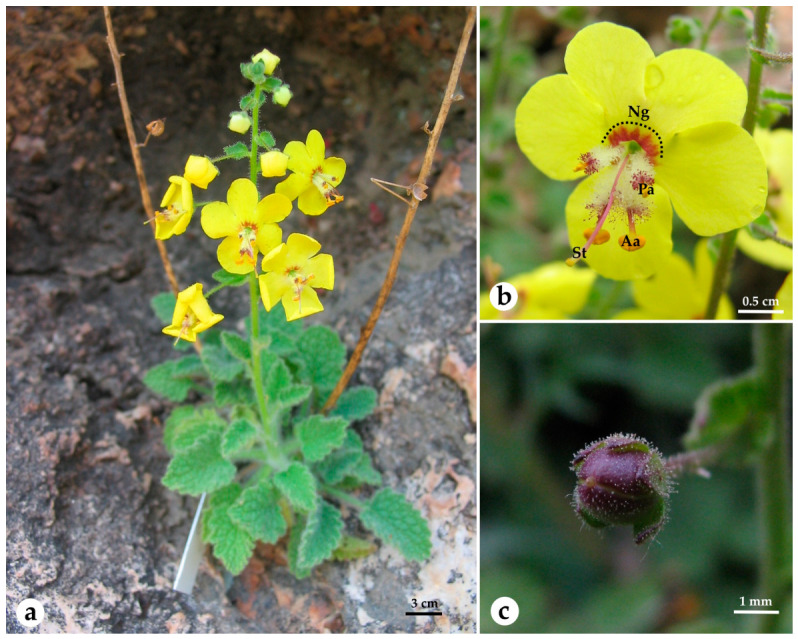
*Verbascum rupicola*. (**a**) habitus, (**b**) floral morphology (St: Style, Aa: Anterior anther, Pa: Posterior anther, Ng: Nectar guidance) (**c**) immature capsule.

*Verbascum rupicola* is a perennial, chasmophytic plant, distributed on vertical hydrothermal quartz vein outcrops and fragmented crevices within the Mediterranean macchia matrix at altitudes of approximately 180–200 m (Figure 4). The species is a steno-endemic with a single known global population located in the vicinity of Gümüldür, within the Tahtalı Dam basin, south of the İzmir city center. Although historical records by Huber-Morath [30] initially cited the type locality as Konya (Koraş) at 2000 m, subsequent floristic investigations and analysis of collectors’ itineraries revealed logistical ambiguities and locality mismatches, confirming the İzmir population as the only extant occurrence of the species [31,60]. The study was carried out between June 2007 and July 2025 in the Tahtalı Dam basin (38°05′25.05′′ N, 27°01′10.83′′ E), which is characterized by a Mediterranean climate and a specific microclimate influenced by the proximity of the reservoir.

### 4.2. Rock Analysis

To characterize the specialized geological substrate of *V. rupicola*, both macroscopic and microscopic analyses were conducted on rock samples collected from the natural habitat. The mineralogical composition and textural features were identified using a polarizing microscope. Transmitted light polarized microscopy was used for transparent minerals, while reflected light ore microscopy was employed for opaque phases using the same integrated optical systems. For microscopic examination, thin sections were prepared by grinding rock fragments to a standard thickness of approximately 0.03 mm. This process involved bonding the sample with Canada balsam onto a slide. Opaque minerals were analyzed using polished sections, which were prepared using diamond abrasives (15–1 µ) to achieve a relief-free surface for reflected light observation. Mineral hardness was determined according to the Mohs scale. Optical properties, including crystal orientation, undulose extinction as an indicator of tectonic pressure, and pseudomorphic transformations, were evaluated. Grain size measurements for quartz and ore minerals were performed using calibrated ocular micrometers under the specified microscope systems.

### 4.3. Distribution Area and Population

Field surveys were conducted between 2007 and 2025 in the Tahtalı Dam basin (İzmir, Türkiye), focusing on the steno-endemic *V. rupicola*. The species’ habitat is strictly confined to specific rocky outcrops along local fault lines. Initially, in 2007, the global population was delineated into four distinct sub-populations (Sp1, Sp2, Sp3, and Sp4). Due to the high visibility of individuals on exposed rocky surfaces and the restricted extent of the habitat, a total census methodology (direct individual counting) was employed. To ensure methodological transparency, it should be noted that field surveys for absolute population size determination were strictly restricted to three discrete census years (2007, 2016, and 2025) rather than continuous annual monitoring.

Spatial boundaries of each sub-population were established by recording field-validated GPS coordinates. These coordinates were imported into Google Earth Pro to delineate the initial Area of Occupancy (AOO) for the baseline year 2007. Vector polygons representing Sp1, Sp2, Sp3, and Sp4 were generated through manual digitization of the distribution limits based on georeferenced field data.

To quantify habitat reduction over the 18-year period, two major disturbance events were mapped through visual interpretation of multi-temporal high-resolution satellite imagery within Google Earth Pro: the spatial extent of a newly established olive orchard within the Sp1 habitat was digitized as a separate vector layer to calculate the area of natural habitat loss (2011), and the boundaries of the catastrophic forest fire affecting the northern and eastern sections of the basin were directly delineated by identifying burn scars and vegetation loss on post-fire satellite imagery (2017).

### 4.4. Style Morph Ratios

**Figure 5:**
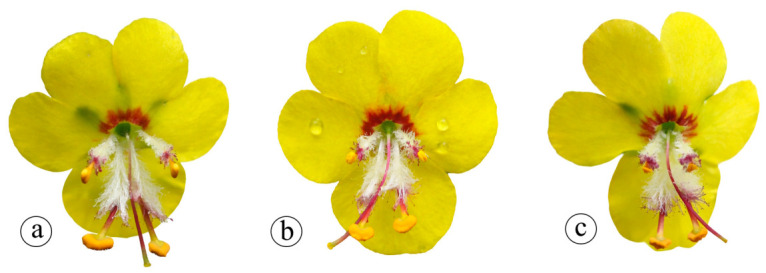
*V. rupicola* flower morphs (**a**) straight tilted, (**b**) left tilted, (**c**) right tilted.

To characterize the floral polymorphism in *V. rupicola*, a morphological survey was conducted across a natural population (n = 50 individuals). For each individual, style orientation was recorded for all flowers on five randomly selected inflorescence axes, proceeding from the base to the apex. Flowers were categorized into three distinct morphs based on style deflection: left-tilted, right-tilted, and straight-styled (Figure 5). A total of 2742 flowers were sampled to evaluate the distribution of these morphs both within the individuals and across the population.

### 4.5. Mating System and Self-Incompatibility Rate

To evaluate the reproductive system, a total of 50 individuals with at least 5 flower axes in Sp1 were labeled in April 2025. For open pollination, apomixis, allogamy, geitonogamy, and autogamy experiments, the 5 flower axes in the individuals were labeled with threads of 5 different colors, and each experiment was performed on a separate flower axis. Each experiment was performed separately on flowers with a straight style, a style tilted to the right, and a style tilted to the left. The following treatments were applied to the flowers of the individuals: (i) For open pollination, flowers were left open for pollinators to visit [n = 50 flowers]. (ii) For the apomixis experiment, stamens were removed and flowers were bagged with waxed paper [n = 50 flowers]. (iii) For the allogamy experiment, the stamens of the flower used as the mother flower were removed and it was pollinated with pollen from different flowers and bagged with waxed paper [n = 50 flowers]. (iv) For the geitonogamy experiment, the flower of the mother plant was pollinated with pollen from different flowers on the same individual and bagged with waxed paper [n = 50 flowers]. (v) For the autogamy experiment, flowers pollinated with the flower’s own pollen were bagged with waxed paper [n = 50 flowers]. Two weeks after the experimental setup was established, capsules were collected and the seeds in each capsule were counted. The total number of ovules was also determined by counting capsules taken from flowers with straight, right, and left styles of marked individuals [n = 50 flowers].

In order to evaluate the breeding system of the species, the self-incompatibility index (ISI) was calculated for left-, right- and straight-styled flowers using the equation proposed by Zapata and Arroyo [61]. The index of self-incompatibility (ISI) was calculated as the ratio of seed set obtained from self-pollination to that obtained from cross-pollination (ISI = selfed seed set/cross-pollinated seed set). According to this classification, an ISI value of 1 indicates complete self-incompatibility, values between >0.2 and <1 indicate partial self-incompatibility, and values < 0.2 indicate mostly self-incompatibility.

### 4.6. Seed Viability, Germination and Seedling Establishment Experiments

To evaluate the physiological quality and germination capacity of *V. rupicola*, a synchronized experimental design was implemented using seeds from Sp1. A total of 1000 morphologically sound and intact seeds were selected and subjected to a two-step surface sterilization: immersion in 70% ethanol for 1 min, followed by 20% sodium hypochlorite for 5 min. After sterilization, seeds were rinsed 3–5 times with sterile water and dried. These seeds were then divided into two equal groups of 500 for parallel assays. For the viability assessment, 500 seeds were distributed into 10 Petri dishes (50 seeds per dish). The seeds were initially imbibed in sterile water for 24 h, then immersed in a 1% 2,3,5-triphenyl tetrazolium chloride (TTC) solution and incubated in darkness at 30 °C for 24 h. Following a brief treatment with 70% ethanol to enhance visibility, embryos were examined under a stereo microscope; seeds with distinct red or pink staining were recorded as viable. Simultaneously, germination assays were conducted on the remaining 500 seeds, also distributed into 10 Petri dishes (50 seeds per dish), and incubated at a constant 20 °C. Germination (radicle emergence ≥ 2 mm) was monitored daily for 10 days until the process reached a plateau.

To assess the establishment capacity of *V. rupicola*, a two-stage acclimatization protocol was implemented. Initially, 60 healthy seedlings derived from the germination assays were transplanted into cell vials filled with a peat-based growth medium. These seedlings were maintained in a growth chamber at a constant temperature of 20 °C with 60% relative humidity. The plants were monitored for three weeks until they reached a developmental stage of 4–8 true leaves, with survival and mortality rates recorded weekly. In the second stage, the surviving individuals were transferred to an outdoor environment to evaluate their adaptation to semi-natural conditions. The seedlings were transplanted into a specialized rock garden at the Ege University Botanical Garden, constructed using rock fragments collected from the species’ native habitats to replicate its chasmophytic environment. Seedling establishment in the rock garden was monitored through weekly observations, recording the survival and success of the transition from controlled to outdoor conditions.

### 4.7. Statistical Analysis

The population-level frequencies of the three floral morphs were analyzed using a Chi-square (χ^2^) goodness-of-fit test. This analysis was employed to determine whether the observed counts of left-, right-, and straight-styled flowers significantly deviated from an expected 1:1:1 ratio. The threshold for statistical significance was set at *p* < 0.05.

Germination data were initially normalized to percentages to standardize the variability between replicates. The kinetics were analyzed using a non-linear regression based on a four-parameter logistic equation (variable slope), with the bottom constraint set to zero. The model allowed for the determination of the maximum germination asymptote (Top), the slope of the curve (HillSlope), and the *EC*_50_ values, all reported with 95% confidence intervals. Statistical fitness was evaluated using the coefficient of determination (*R*^2^). A paired *t*-test was applied to evaluate the relationship between seed viability and germination success. The correlation between viability and germination was assessed using the Pearson correlation coefficient (r).

To account for the non-independence of multiple flower axes sampled from the same individual, reproductive data were analyzed using a Linear Mixed-Effects Model (LMM). In this model, ‘Individual Plant’ was treated as a random factor, while ‘Pollination Treatment’ and ‘Style Position’ were designated as fixed factors. Following the LMM, Tukey’s multiple comparison test was applied to evaluate significant differences between treatments.

To evaluate the influence of environmental factors on flower productivity and flower predation rate, a Multiple Linear Regression (MLR) model was constructed using individual floral counts and predated flower rate (N = 300) from the 2007, 2016, and 2025 census years. Meteorological data, including mean temperature (°C) and total precipitation (mm), were obtained from the Turkish State Meteorological Service. Mean temperature (°C) and total precipitation (mm) during the peak flowering months (March and April) were used as independent predictor variables. Model assumptions were rigorously tested; multicollinearity was assessed via the Variance Inflation Factor (VIF), and the normality of residuals was evaluated using Shapiro–Wilk and D’Agostino–Pearson omnibus tests.

The threshold for statistical significance for all tests was set at *p* < 0.05. All statistical fitness was evaluated using the coefficient of determination (*R*^2^), and analyses were conducted using GraphPad Prism 8.4.2.

### 4.8. IUCN Conservation Status Assessment

The conservation status of *V. rupicola* was evaluated following the IUCN Red List Categories and Criteria (Version 3.1). This evaluation integrated the 18-year longitudinal quantitative data, specifically focusing on the temporal reduction in the Area of Occupancy (AOO) and the severe decline in the number of mature individuals resulting from habitat fragmentation and stochastic fire events. These field-derived spatial and demographic parameters were systematically assessed against IUCN Criteria B (Geographic range) and C (Small population size and decline) to determine the species’ extinction risk category.

## 5. Conclusions

In conclusion, based on the formal assessment of our 18-year spatiotemporal and demographic data, *V. rupicola* meets the requirements for Critically Endangered (CR) status under the IUCN criteria B1ab(i, ii, iii, iv, v) + 2ab(i, ii, iii, iv, v). Our findings suggest that ex situ conservation efforts, such as seed gene banking, are insufficient on their own to ensure the species’ long-term survival. The most effective strategy for preventing total extinction lies in the absolute protection of the Sp1 habitat as a ‘micro-reserve’ and the immediate initiation of in situ restoration trials within stabilized post-fire areas. Protecting this unique geological niche is not merely a local necessity but a global imperative for preserving Mediterranean plant biodiversity.

## Figures and Tables

**Table 1:** Spatial and demographic changes in *V. rupicola* sub-populations (2007–2025).

Sub-Population	2007 Area (m^2^)	2007 Individuals	2025 Area (m^2^)	2025 Individuals
**Sp1**	298,801	810	198,973	386
**Sp2**	134,621	183	0	0
**Sp3**	142,433	193	0	0
**Sp4**	47,919	64	0	0
**Total**	623,765	1250	198,973	386

**Table 2:** IUCN Red List assessment for *V. rupicola* based on 18-year monitoring data.

Criterion	Parameter	Value/Status	Justification and Calculation Path
B1	EOO (Extent of Occurrence)	<1 km^2^	Total range is restricted to a single locality in the Tahtalı Dam basin. Even with a minimum convex polygon, it is far below the CR threshold (<100 km^2^).
B2	AOO (Area of Occupancy)	0.199 km^2^	Calculated from georeferenced field data (198,973 m^2^). Far below the CR threshold (<10 km^2^).
B1a/B2a	Number of locations	1	Only one surviving sub-population (Sp1) remains; a single event (e.g., fire or agricultural expansion) could drive the species to extinction.
B1b/B2b (i–iv)	Continuing decline	Observed	Documented decline in: (i) EOO, (ii) AOO, (iii) habitat quality/area, (iv) number of locations, and (v) number of mature individuals (69.12% collapse).
Final Status	CR	Critically Endangered	B1ab(i, ii, iii, iv, v) + 2ab(i, ii, iii, iv, v).

## Data Availability

The data is archived at the Ege University Botanical Garden and Herbarium Research and Application Center and is available upon request.

## Supplementary Materials

The following supporting information can be downloaded at: https://www.mdpi.com/article/10.3390/plants15101555/s1, Figure S1: Comparison of *V. rupicola* habitat (Subpopulation 1) in the Tahtalı Dam basin: (a) intact habitat before disturbance in 2007; (b) habitat degradation and substrate loss after anthropogenic disturbance in 2025; Figure S2: The *Pieris rapae* caterpillar, a predator of *V. rupicola*, and the damage it causes; Figure S3: Ex situ cultivation phases of *V. rupicola*: (a) young seedlings in multi-cell trays; (b) juvenile individual transplanted to the garden, showing bud formation; (c) full flowering stage; File S1: *Verbascum rupicola* all data.

## Abbreviations

The following abbreviations are used in this manuscript:

Sp	Subpopulation
ISI	Index of Self-Incompatibility
IUCN	International Union for Conservation of Nature
