# Supplementary figures and images for: 18-Year Monitoring of the Steno-Endemic Verbascum rupicola (Scrophulariaceae): Compounding Pressures and the Extinction Vortex
Source: Plants (Basel). 2026 May 20;15(10):1555. doi: 10.3390/plants15101555 (PMC13210554; doi:10.3390/plants15101555)

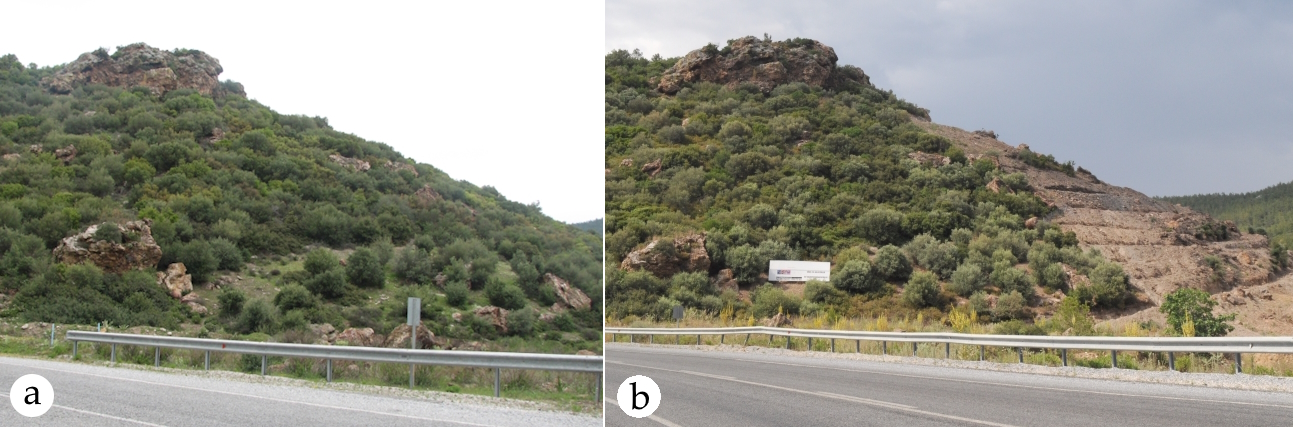

Supplement: Supplementary file 1 [file plants-15-01555-s001.zip › Figure S1.jpg]

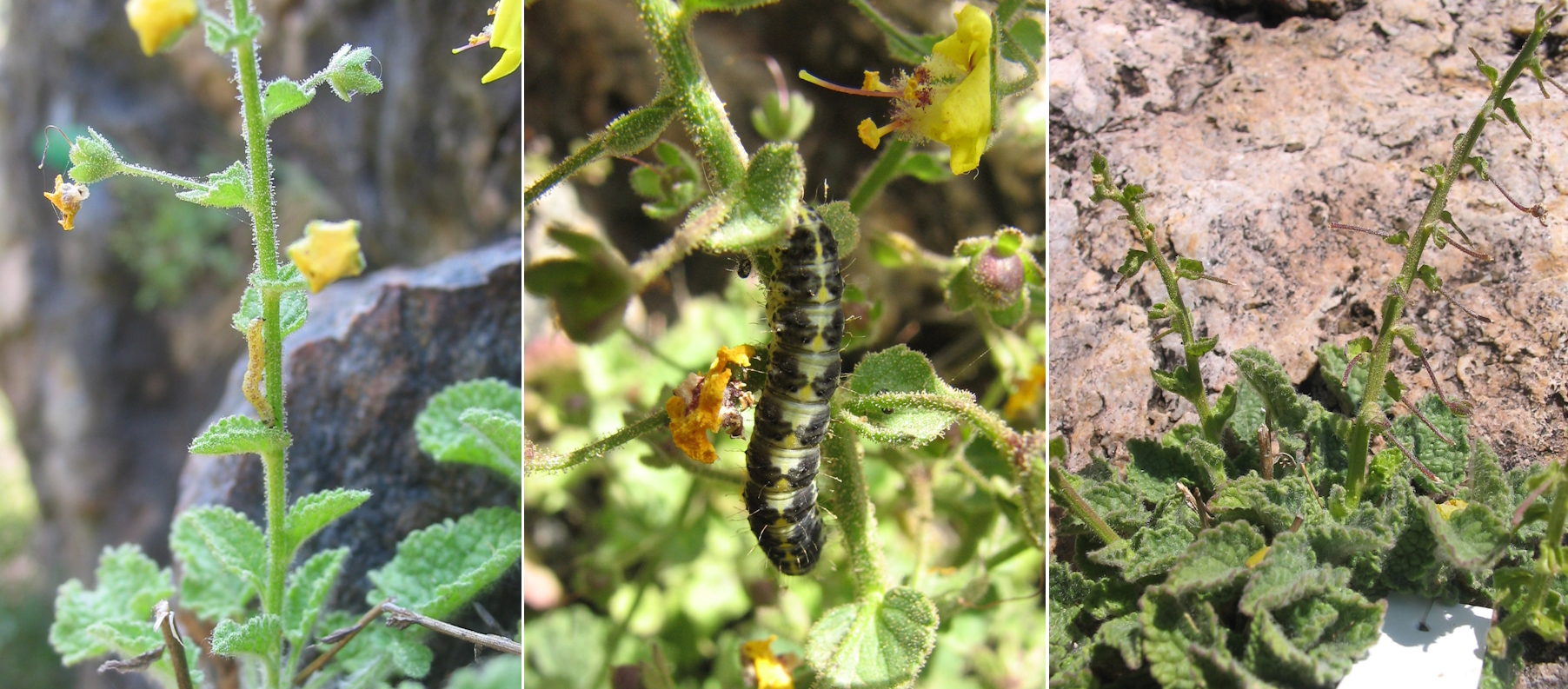

Supplement: Supplementary file 1 [file plants-15-01555-s001.zip › Figure S2.jpg]

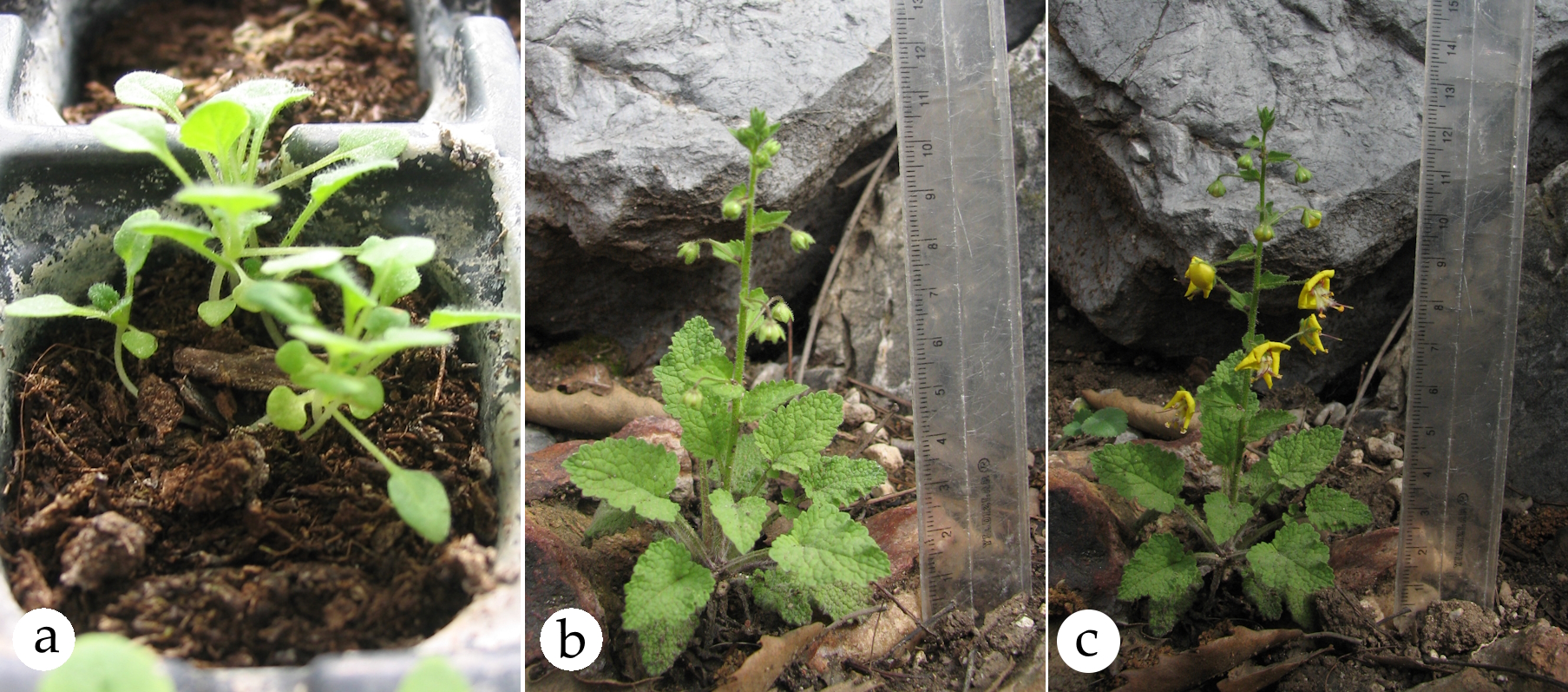

Supplement: Supplementary file 1 [file plants-15-01555-s001.zip › Figure S3.jpg]
